# Active2Gether: A Personalized m-Health Intervention to Encourage Physical Activity

**DOI:** 10.3390/s17061436

**Published:** 2017-06-19

**Authors:** Michel C. A. Klein, Adnan Manzoor, Julia S. Mollee

**Affiliations:** Department of Computer Science, Vrije Universiteit Amsterdam, De Boelelaan 1081, 1081 HV Amsterdam, The Netherlands; m.c.a.klein@vu.nl (M.C.A.K.); a.manzoor@vu.nl (A.M.)

**Keywords:** e-coaching, m-health intervention, personalization, healthy lifestyle, physical activity

## Abstract

Lack of physical activity is an increasingly important health risk. Modern mobile technology, such as smartphones and digital measurement devices, provides new opportunities to tackle physical inactivity. This paper describes the design of a system that aims to encourage young adults to be more physically active. The system monitors the user’s behavior, uses social comparison and provides tailored and personalized feedback based on intelligent reasoning mechanisms. As the name suggests, social processes play an important role in the Active2Gether system. The design choices and functioning of the system are described in detail. Based on the experiences with the development and deployment of the system, a number of lessons learnt are provided and suggestions are proposed for improvements in future developments.

## 1. Introduction

Physical inactivity is an increasingly serious health problem: the World Health Organization (WHO) has identified that it is the fourth leading risk factor for global mortality [1]. The organization estimates that a lack of physical activity leads to 3.2 million deaths per year globally. Physical inactivity has all to do with modern sedentary lifestyles, which are led by 60% to 85% of people worldwide, according to the WHO. One aspect of a sedentary lifestyle is that people are more inclined to passive modes of transportation. Active travelling modes such as biking and walking can contribute to a healthy level of physical activity [2]. Another aspect of a sedentary lifestyle is related to the work environment, where much work is done by people seated in chairs in front of computers. Research suggests that having desk jobs increases health risks up to 50%. Integrating small activities in work routines can help to increase physical activity and lower health risks [3]. 

At the same time, modern mobile technology, such as smartphones and digital measurement devices, provides new opportunities to tackle physical inactivity. In 2015, 43% of the adults worldwide owned a smartphone, with percentages up to 70% for developed countries [4]. Smartphones allow for continuous and real-time monitoring of activity behavior via built-in sensors such as accelerometers, and provide possibilities for giving contextualized and personalized feedback. This makes the smartphone a potentially powerful device for real-time coaching of people towards a more active lifestyle. To use smartphones and sensors for this aim, they should be integrated into a behavior change support system, which is defined as an information system designed to form, alter, or reinforce attitudes or behaviors [5]. 

In this paper, we describe the design of such a system in detail, together with lessons learnt and suggestions for future developments. The system is developed in context of an interdisciplinary research project and is called Active2Gether. The goal of the project is to combine domain knowledge from experts in physical activity interventions with modern mobile technology to design an intervention that encourages physical activity among healthy young adults. One of the innovative aspects of the system is that it exploits model-based reasoning techniques for tailoring the coaching to the needs of the user. Up to now, this has hardly been applied within existing interventions [6].

As the name suggests, social processes play an important role in the Active2Gether system. This is reflected in different ways, such as the implementation of social comparison mechanisms on both an individual and a group level. In addition, the system addresses psychological constructs as social norms and social aspects of outcome expectations in its coaching messages.

The aim of the Active2Gether system is to increase or maintain levels of physical activity among young adults in the age group of 18 to 30 years. The system is being evaluated in a trial (see [7] for a detailed description) in which over 100 participants, aged between 18 and 30 years old, used either a variant of the Active2Gether system or the standard website that belongs to a commercial activity tracker for approximately three months. The user evaluation of the system by the participants is described in [8]; in this paper, we focus on the architecture and functionality of the system.

## 2. System Description

To the user, the Active2Gether system presents itself as an Android-based mobile phone app that continuously monitors the context of a person. One of the distinct features of the system is that it implements evidence-based behavior change techniques, unlike most apps that are currently available in the app stores [9]. The most promising behavior change techniques are employed in the app, including self-monitoring, performance feedback, goal setting and social comparison.

The app performs four main functions: it communicates with the user about his/her objectives regarding physical activity for the next week, provides timely and personalized feedback, facilitates self-monitoring based on several collected data sources, and supports social comparison with the help of Facebook friendship relations. The system focuses on three types of physical activity: leisure time sports activities, active transport and stair walking. Users can choose to be coached on at most one of these three domains at the same time.

In the following sections, we describe the design of the system in detail. We first describe the data that are collected by the system. We then provide an overview of the architecture of the system and describe the layout choices. After that, we explain the working of the reasoning engine and the selection and filtering of coaching messages. Finally, we describe the implementation of the social comparison functionality.

### 2.1. Data Collection

The system uses several mechanisms to collect information from and about the users. Below, we describe how questionnaires as well as sensor measurements are used to understand the user behavior.

#### 2.1.1. Intake Questionnaires

The user starts with filling in an online intake questionnaire. Besides demographic information, this questionnaire asks about their significant locations (such as home, work, study, etc.), their travel options between these locations, and psychological factors underlying their physical activity behavior (e.g., skills, barriers, goals, and outcome expectations). The answers to these questions are used for tailoring the messages to the user’s personal situation and are used in the model-based reasoning about the effect of specific coaching strategies on a specific user (see Section 2.4).

#### 2.1.2. Activity Tracker

The commercial Fitbit One is used as activity tracker that registers the daily number of steps and the number of stairs climbed [10]. Users receive an account on the Fitbit website. The Fitbit device uses Bluetooth LE to automatically synchronize activity data with the Fitbit servers, either via the Fitbit mobile phone app, or via a Bluetooth LE dongle and a pc. After the first login to the Active2Gether system, the user is asked to connect to his/her Fitbit account with the Active2Gether system. A connection is established through an open authentication mechanism. Once the connection is made, Fitbit provides an authentication key for the user. This key is stored in the database, so the Active2Gether system can directly access the Fitbit web service to receive activity data for a user and store it into the Active2Gether database. A script runs every hour to update the database with the most recent activity data. Another script periodically checks whether the battery level is low or whether the last synchronization is more than three days ago, and, in that case, a reminder is sent.

#### 2.1.3. GPS Location

The Active2Gether app uses the built-in GPS sensor for recording the GPS coordinates (latitude and longitude). As soon as a user logs in, he is asked to authorize the use of location tracking. It is possible for a user to turn off the location detection option, but this will disable certain features. In a separate experiment, we compared different time intervals for collecting GPS data [11]. It turned out that a frequency of five minutes provides a good balance between battery consumption and precision. Every 15 min, the data on the mobile phone are synchronized with the server. In the database, latitude, longitude, speed, accuracy and time stamp are stored for each observation. 

A script runs every night to see whether a user has visited one of his important locations by comparing the GPS trace with the coordinates of the important locations. Since the user locations are provided in descriptive form, geocoding is used to transform them into latitude and longitude numbers. The system stores the number of minutes at each of the locations. If the duration at a location is larger than 0 min, we can conclude that the user visited that location.

#### 2.1.4. Daily Questions

Every day, a number of questions is posed to the user via the app. Information about the visited locations is used to prompt the user about the travel options that he used to go there. As the users had to list two options (active and inactive) in the intake questionnaire, they are asked to choose between these two options. Since the system is aware of the activity level of the different options for each user, it can derive the types of transport used and the amount of active travel minutes.

In addition, the user is prompted about the sports activities during the day before. When a user regularly answers that he did not participate in sports activities, the frequency of asking about sports is decreased to once per week.

### 2.2. Architecture

**Figure 1:**
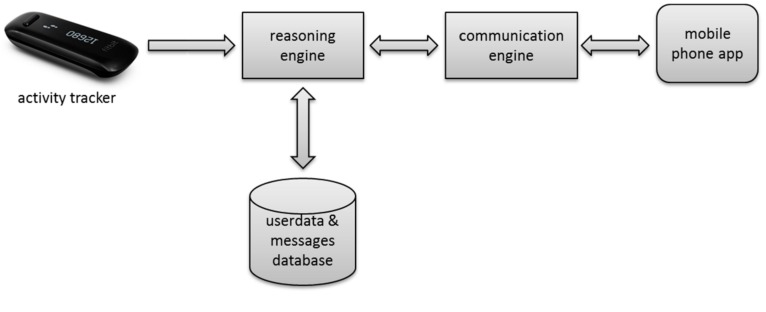
Overall architecture of the Active2Gether system.

The system is comprised of five main components: (1) an app on a mobile phone; (2) a commercial activity tracker; (3) a database with user (activity) data and persuasive messages; (4) a model-based reasoning engine to interpret the data and predict the effect of different coaching strategies; and (5) a communication engine that selects and sends questions and messages to the app. Figure 1 shows those main components.

**Figure 2:**
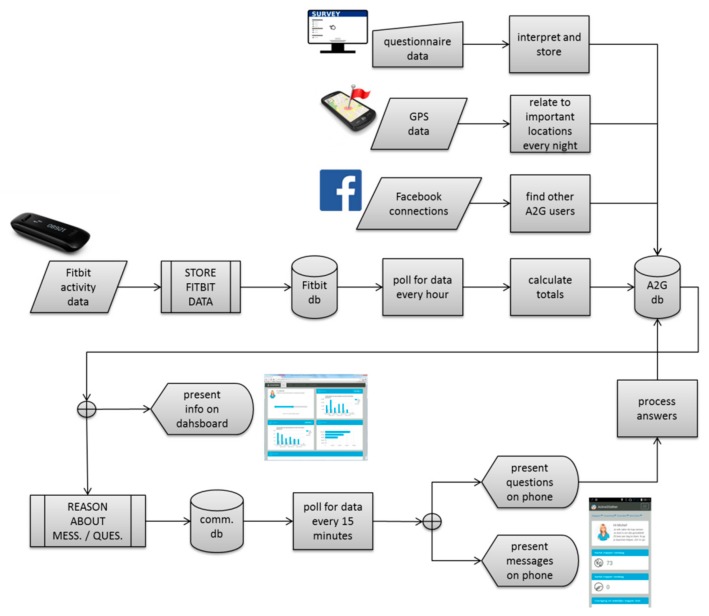
Overall data flow in the Active2Gether system.

Figure 2 provides an overview of the most important data flows in the system. The details are provided in the following sections.

#### Structure of the App

In order to provide users the possibility to also view their information via a website, the main dashboard of the system is developed as a web page. Within the app, the main component is a GUI element (i.e., a WebView component) that renders this web-based dashboard. Since a responsive web design approach is followed, the website automatically adapts to smaller screen sizes. Although the dashboard is actually a website, users do not notice this. The app behaves like a native app and users do not need to login separately via the WebView: once a user has registered his/her account for the Active2Gether system in the Android system, the app uses those credentials to automatically log in the user and to show the appropriate page inside the WebView component as if it is a screen in the app itself.

The other functions of the mobile phone app are to facilitate the communication with the user and to monitor the user’s location. The latter is done with the help of Google location services. Using the built-in Android synchronization system, the app connects every 15 min via a web service to the communication engine of the system. Messages or questions that are prepared for the user by the reasoning engine are collected and answers and read notifications are sent back. Whenever a new message or question is sent to a user, it appears in the status bar and when the user clicks on the message, it is shown as an overlay on the main screen.

### 2.3. Layout and Visual Design

In order to show a consistent look and feel to the user, a professional designer was hired to design and recommend different aspects of the user interface. The designer helped in suggesting layout, fonts and a coloring scheme for the website and consequently the dashboard of the app. There are eight panels (small rectangular windows) on the website, which show different kinds of information to the user depending on the chosen coaching domain for the current week. The first panel shows a picture of the coach and a welcome message corresponding to the current coaching domain. For example, if the current coaching domain is active transportation, the message is: *“Hi Adnan! You have chosen to focus on active transportation this week. Your goal is to spend this week at least 36 minutes of active transportation. I will support your efforts.”* The activity data are presented in many different views. Two small panels show the most recent steps and floors count for the present day. Whenever a user visits the website or opens the app, a dynamic script runs to show a summary of the most recent data in the Active2Gether database.

**Figure 3:**
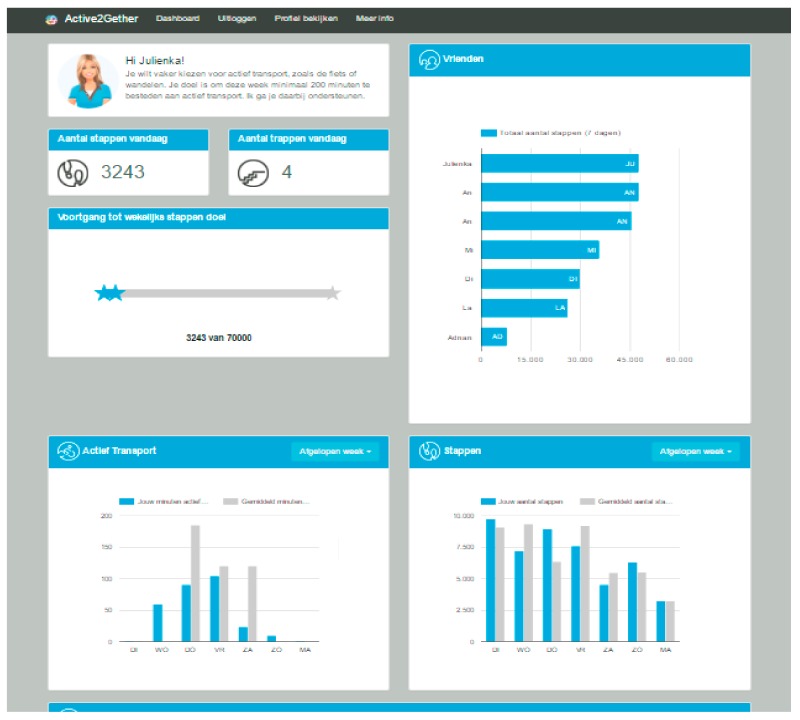
Screenshot of an Active2Gether dashboard.

A panel with the caption “Progress to weekly step goal” shows a progress bar towards a weekly goal of 70,000 steps. There is another panel that shows the performance of other users (see Section 2.6 about social comparison below). Another panel shows the type of physical activity based on the chosen domain for the current week (active transport, stair walking or sports activities). In the first week, when no domain has been chosen yet, this panel shows the number of active minutes based on the reported sports activities and active travel choices. A similar panel shows the user’s activity in terms of the number of steps. The latter two panels provide an option to the user to view historical data per week, per month or from the beginning. This option is useful for those users who want to see their own past performances. They also provide the user an opportunity to compare his/her performance with the average values of all users. The final panel is dedicated to show the most recent messages. Figure 3 gives an example of the dashboard.

### 2.4. Model-Based Reasoning

**Figure 4:**
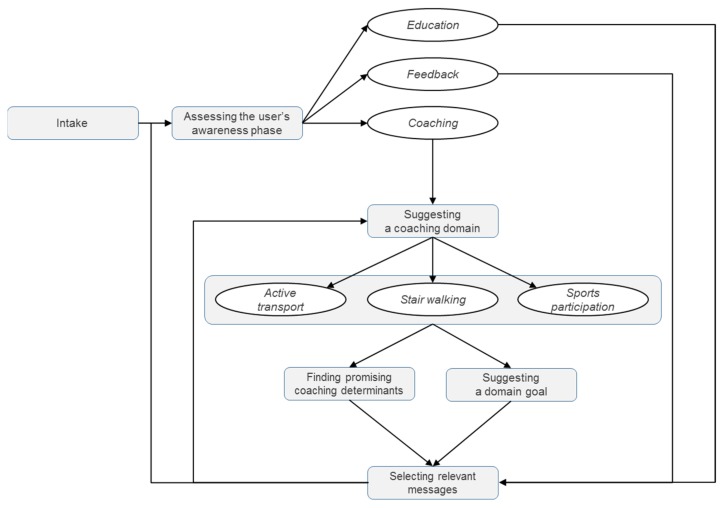
Process flow chart of reasoning engine.

One of the fundamental components of the Active2Gether system is the so-called reasoning engine, which analyzes and interprets the user’s data and determines what type of support the user should receive. A core component of this reasoning engine is a computational model, which is discussed below. The reasoning process can be split up into three parts: assessing the user’s activity and awareness level, suggesting a coaching domain based on hypothesized room for improvement, and predicting the most promising coaching determinants. Figure 4 shows a flow chart of the processes taking place in the reasoning engine.

#### 2.4.1. Assessing the User’s Awareness Phase

In the first step of the reasoning engine, the user’s current activity level and awareness phase are assessed to determine what type of support they need. The assessment is based on two evaluations of the user’s physical activity level, namely an objective evaluation (i.e., whether the user meets the norm) and a subjective evaluation (i.e., whether the user thinks he/she is sufficiently physically active). Using that information, the user is assigned to one of four categories, each representing an awareness state regarding their physical activity. The resulting categories are summarized in Table 1. Category 1, in which users believe that they are sufficiently active but objectively do not meet the norm, is more or less comparable with the *precontemplation* phase in Prochaska’s Transtheoretical Model of behavior change [12]. In this phase, people are uninformed or under informed about the consequences of their behavior and education about the consequences is needed. Category 4 is similar to the *maintenance* phase, while Categories 2 and 3 are comparable to the *action* phase in Prochaska’s model. Because our system can objectively measure whether people meet the norm, we can determine an awareness phase in a simpler and more accurate way than with the questions that are often used for determining the stage according to the transtheoretical model, and without the strong assumption that people always go through all phases.

Based on the categorization of the awareness of users, the system determines which type of support (i.e., *education*, *coaching* or *feedback*) the user needs. This assessment is reflected in the type of motivational messages that the user receives from the app (see also Section 2.5). In addition, for users who receive coaching, the system guides them to choose a coaching domain, prompts them to set a specific goal, and predicts the most promising coaching determinants, as further explained below. This evaluation is repeated every three weeks, in order to continuously tailor the system to the user’s current state. Thus, instead of treating all users the same, their specific needs and wishes are taken into account. This should lead to improved user acceptance and adherence, and consequently to increased effectiveness of the intervention [13].

#### 2.4.2. Suggesting a Coaching Domain and Goal

Users that are assigned to the coaching category are guided by the Active2Gether system to focus their behavior change efforts, by advising on the choice of a specific coaching domain and a goal. This cycle is repeated on a weekly basis. The coaching domains are parts of the user’s daily life: (1) stair use at significant locations (e.g., home, work, and university); (2) active transport to significant locations; and (3) leisure-time sports activities.

First, detailed information about the user’s context and behavior is used to identify in which domains the user could be more physically active. The user’s physical activity in each of the three domains is estimated based on a combination of activity data collected through the activity monitor (number of stairs climbed) and daily user input through the app (selected transport options to visited locations, time spent on sports activities). These physical activity values are then evaluated by comparing them to estimated “maximum” or “ideal” values, which are based on information about the user’s context and visits to their important locations. This context information is collected through an intake questionnaire, and includes information about the addresses of their significant locations, (active and non-active) travel options between these locations, relevant floor numbers on these locations, and the availability of stairs. For example, if a user works on the third floor and on average climbs another three floors during the day, a total number of six floors during a work day would be reasonable. For a user that works on the second floor, but on average climbs another eight floors during a work day, a total number of six floors is comparatively low. In addition, the more often the user has gone to work, the higher the expected number of stairs becomes. Similar evaluations are developed for the physical activity level in active transport and sports activities. Using these evaluations, the domain with the largest potential for improvement can be detected, as the evaluation score for that domain will be lowest. This domain is then suggested to the user as focus for the coaching in the upcoming week. However, the user is allowed to overrule this suggestion and opt for another domain.

After selection of a coaching domain, the user is asked to set a specific goal for this coaching domain, i.e., weekly time spent on active transport, weekly time spent on sports or daily number of stairs climbed. If users did meet the previous goal in this coaching domain, the system suggests increasing their goal by 10%. If users did not meet the goal last time, the system advises to keep the goal at the same level. Again, to ensure the user’s autonomy, the final decision on the goal is up to the user.

This relative evaluation of the user’s behavior and the recommendation of a certain coaching domain respect the individuality of the users more than general physical activity guidelines. It prevents the system from imposing the same expectations on all users, even though their personal situations may be completely different.

#### 2.4.3. Finding the Most Promising Coaching Determinants

Once the coaching domain is selected, the system investigates on which personal determinants the coaching messages should focus to yield the most promising effect on the desired behavior. These behavioral determinants are personal psychological concepts that govern the engagement in healthy behavior. The system contains a large collection of coaching messages, with subsets that each target one of the personal determinants. The messages are based on established behavior change techniques, such as prompting barrier identification, providing information on consequences, and prompting goal setting [7].

**Figure 5:**
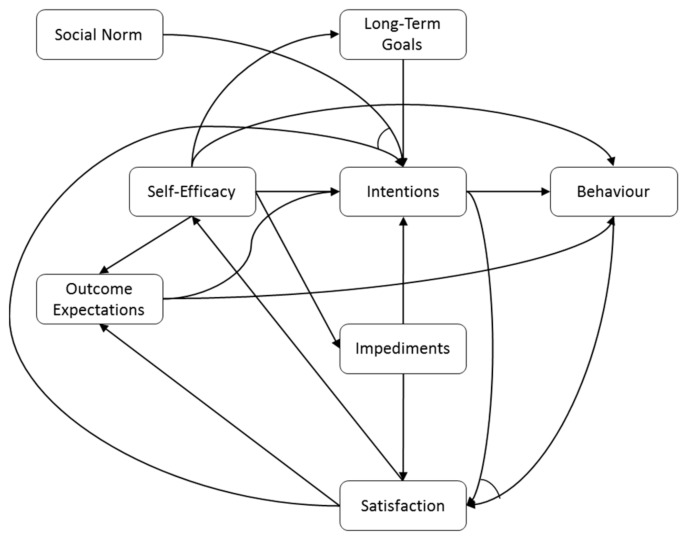
Graphical representation of the computational model.

In order to determine what messages are most likely to positively affect the user’s behavior, the effects of improving each one of the personal determinants are estimated based on simulations of a dynamic computational model. This model is a formalization of the dynamics between these personal determinants and the behavior, where each of the concepts is represented by a numerical value in range [0, 1]. The model is mainly based on the social cognitive theory, that describes the reasons why people fail or succeed to exhibit some desired (health) behavior from both social and cognitive determinants [14,15]. Other theories (e.g., self-regulation theory and health action process approach) and literature were consulted to extend the model to incorporate more relevant aspects. The model that was implemented in the Active2Gether system is an adaptation of the computational model presented in [16]. Revisions between the two versions of the model were motivated by a decrease in conceptual detail and computational complexity of the model, and by suggestions of experts in the domain of behavior change. The resulting model contains determinants such as intentions, self-efficacy and outcome expectations. The values of all parameters in the model can be adjusted by the modeler. In the current implementation, the parameters were chosen based on correlations between the concepts found in literature [17,18,19]. We performed simulations to find values that keep the ratio between the parameters in accordance with empirical findings [20]. A more detailed description and a preliminary validation of this model on empirical data, which showed promising results, can be found in [21]. Figure 5 shows a graphical representation of the computational model.

The simulation process starts with estimating the current states of the personal determinants by means of short questions via the app. The resulting values are used as input for the computational model. To simulate the effect of targeting one of the determinants, one of the values obtained from the app questionnaire is increased according to the hypothesized effect of sending coaching messages about this determinant. Then, the computational model simulates the dynamics between the determinants and estimates the effect on the behavior. By running simulations for each possible targeted determinant, a list of determinants is constructed, ordered by the most promising effect on the behavior variable. This order is taken into account when selecting coaching messages to the user. As with the selection of a coaching domain and goal, the simulation cycle is repeated weekly, in order to tailor to the user’s strongest psychological needs at all times.

In contrast to the relative evaluation of the user’s behavior for suggesting a coaching domain, this part of the reasoning engine does not tailor the intervention based on information about the user’s environment, but rather on information about his/her motivational state of mind. This way, the users will receive support on the aspects that are relevant to their motivation and behavior.

### 2.5. Selection and Tailoring of Coaching Messages

Once the order of the most promising coaching strategies is established (as described in Section 2.4.3), all necessary elements to send the coaching messages are in place. As explained before, each coaching strategy represents a set of messages targeting one of the concepts from the computational model. The messages are based on established behavior change techniques (e.g., prompting specific goal setting, time management; see [22] for a complete taxonomy), while also taking into account user preferences [23]. They were written to be motivational, personally relevant and trustworthy, and were annotated with restrictions for the circumstances under which they are relevant (e.g., day and time, the user’s awareness phase and coaching domain, the user’s perceptions reported in the intake questionnaire). A detailed description of the techniques that are applied in the messages and the related determinants is provided in [7]. The messages are sent up to three times a day. In order to send a message, it has to be selected from the set of available messages and (if applicable) tailored to the user.

At given moments in time, the communication engine of the system checks for messages that are relevant to send to the user. Selecting a message is based on elimination: starting from the set of all messages, the selection is narrowed down by filtering inapt or irrelevant messages. As explained in Section 2.4, the user is already assigned an awareness phase and has chosen a coaching domain. In addition, a coaching determinant is picked from the ordered list, with a probability relative to its position in the list. This probability is introduced to increase diversity in the messages during the week. Then, all messages that are aimed at other awareness phases, coaching domains or targeted coaching determinants are filtered out. The system checks for other aspects of the message’s relevance as well, such as the day and time, the user’s occupational status, answers to questions in the intake questionnaire, whether the user is on track to reach their goal and the current weather.

Once a message is selected, if there are open fields, they have to be filled in to tailor the message to the user and context. In order to increase relatedness, most messages address the user with their first name. Additionally, some messages are completed by filling out the user’s current daily number of steps or stairs, their accumulated weekly time spent on sports or active transport, their weekly goal, the percentage of their weekly goal they have reached so far, the maximum number of stairs they would consider to walk, or the current weather score.

The coaching strategies are only relevant if the user is in the coaching phase (see Section 2.4), as only then certain determinants from the computational model are targeted. However, also when users are in the education phase or in the feedback phase, they receive messages on a regular basis. In the education phase, users receive messages that put their (insufficient) performance into perspective, as well as messages that emphasize the need for and benefits of physical activity. In the feedback phase, users receive positive feedback on their satisfactory behavior. In addition, users in all awareness phases may receive general messages, regarding their current daily step or stairs count, containing universal motivational phrases, or to remind them to charge their Fitbit or to synchronize their data.

### 2.6. Social Comparison

The Active2Gether system uses social comparison, as one of the core ideas behind the system is that a healthy lifestyle can be maintained and achieved in the presence of social support network. Social comparison is implemented in two ways in the Active2Gether system, namely on a group level and on an individual level. On the group level, social comparison is implemented by showing group averages adjacent to the user’s physical activity data in the graphs on the dashboard. This allows the user to compare his/her daily performance to other Active2Gether users anonymously. 

On the individual level, social comparison is implemented as a ranking of the user’s performance within a list of other users, which is shown in one of the panels in the app/website. The ranking automatically updates every time a user visits the website or opens the app. Overall physical activity is used as basis for the comparison, which is determined by the number of steps taken by an individual in the last seven days, but implementations based on activity data for one of the coaching domains are also conceivable. 

In order to increase the relevance of the comparative data, the system tries to show actual friends of the user in the ranking. To do so, it extracts friendship relations from Facebook. Facebook is an obvious choice, since it is one of the most popular social media websites globally and also very popular among Dutch young adults. A connection to the Facebook Graph API is established through an open authentication mechanism. As a new user logs in for the first time on the system, it asks the user whether he/she wants to grant access to Active2Gether to check for friendships with other Active2Gether users. If permission is granted and a match is found, the friendship connection is also registered in the Active2Gether system. It is not mandatory for users to grant access, but individuals who do not opt for such explicit social comparison can only observe the activities of other users anonymously, which will probably make the social comparison less effective.

Social comparison can be either *upward* or *downward*, depending on whether an individual compares to targets that perform better or worse. Both variants address different underlying motivational processes. Upward comparison can be beneficial if individuals use the target as a role model and motivation to self-improve, but it can have a discouraging effect if the target’s performance seems unattainable [24,25]. Downward comparison can boost an individual’s self-esteem and thereby lay the groundwork for self-improvement [26]. However, downward comparison could also have an adverse effect, since it leads to relatively low goals and since it does not challenge an individual to minimize the discrepancy with a better performing individual. An experiment testing the effects of presenting people with their preferred or opposite direction of social comparison in the domain of physical activity showed that it is important to take personal preferences into account [27]. Participants who were shown the type of social comparison opposite to their preference showed a decrease in overall physical activity. Showing the preferred direction of social comparison typically resulted in an upward trend, which was not statistically significant however. Even though the preferences were based on a simple question (whether the participants prefer to compare themselves to individuals who perform better or worse) and the sample size was small, the results demonstrated that these preferences matter: if not to enhance the motivational effects of social comparison, then at least to avoid the potential adverse effects of social comparison [27].

**Figure 6:**
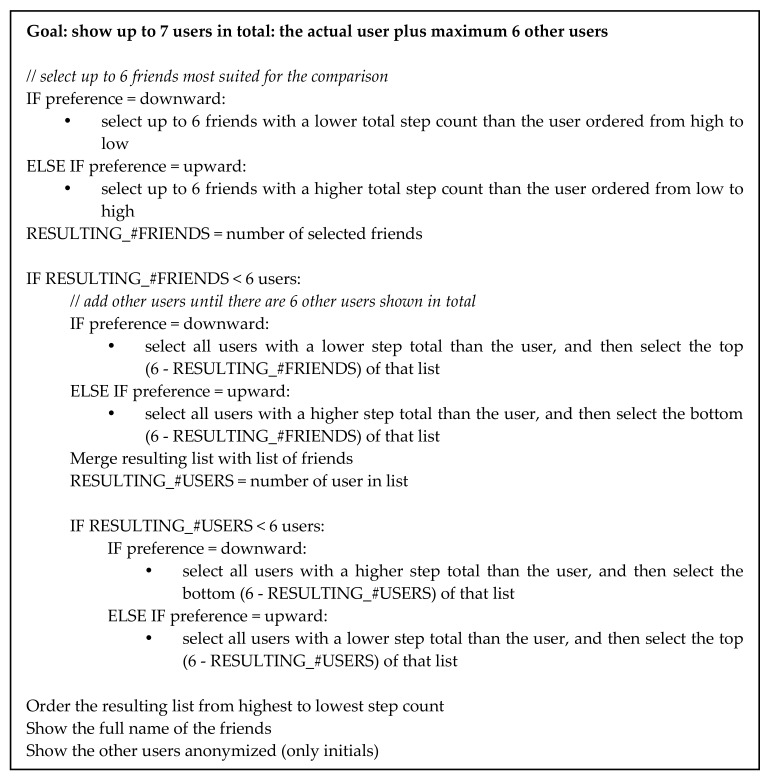
Algorithm for selecting users for the social comparison.

When creating the ranking, the Active2Gether system takes the social comparison preference (upward or downward) of the users into account. The comparison preference of users was determined in the intake questionnaire with the same question as in the experiment described above [27]. The system first tries to find up to six friends whose activity level is in line with the preferred comparison direction. If there are more than six friends that match the preferred direction, the six friends that are closest to the current user in terms of step total of the last seven days are selected. If there are less than six friends in the preferred direction, the system selects other users in the preferred direction. If not sufficient people are found to create the ranking, then the system searches for users in the opposite direction, since it is not appropriate to show an empty ranking list to the user. This could happen, for example, if an individual’s preference for social comparison is upward, but his performance is among the best of the users. The data of befriended users are shown with their first name, but data of other users are shown with their initials only, to maintain a level of anonymity. Figure 6 shows the step-by-step process of selecting the friends or other users to show in the ranking.

## 3. Discussion

In this section, we offer suggestions for improvements based on our experiences with the development and use of the current Active2Gether system.

### 3.1. Data Collection

Advances in (mobile) technology open up ways to improve the location and travel monitoring in the Active2Gether system.

First, for determining modes of transportation, we currently use daily questions in combination with location data (i.e., prompted user input). At the time of the design, this was a reasonable choice, considering the state-of-the-art and the consequences on battery consumption during full-time use of accelerometers in the mobile phone. Nowadays, the Google Activity Recognition API and the iOS CM Motion Activity class would be logical candidates, as they are the de-facto standards for providing location information [28,29]. Power consumption remains an important issue, however.

Second, we used a complicated questionnaire for reporting significant locations and their characteristics (such as relevant floors). It is difficult and cumbersome to answer, and also difficult to update during the intervention. Therefore, we recommend to automatically detect significant locations [11], which can also be done via Google location services. A remaining drawback, however, is that the users are required to provide this privacy-sensitive information.

### 3.2. Architecture

The decision to use a combination of a web-based approach and a native app, which requires more-or-less permanent Internet connection, turned out well. Most users in the Netherlands apparently have good Internet connections on their phones. A drawback of our current choice was that integration with third-party APIs (i.e., Fitbit and Facebook authentication) was difficult, since Fitbit does not allow using their authentication API through the WebView. This can be solved with the newer Chrome tabs approach. 

We decided to copy the data from Fitbit servers to our own database: a Cron job runs periodically to fetch the data through a web service. The advantage is that we could do our analyses more easily (e.g., summarizing the data for different time periods every hour) and have a good performance when we query the data. A disadvantage is that the information sometimes lags behind. If the performance is sufficient, we would recommend to dynamically invoke a web service at runtime. Another possible solution is the use of the use of more advanced services, such as the Fitbit subscription API, which allows sending notification to our system when new data are available. 

Related to the point above, we let participants use the Fitbit app to synchronize their data with the activity tracker. This was necessary because it is not possible to read out the Fitbit device directly. As a consequence, the Fitbit app or a computer was needed in addition to our own system. It also required an additional step in the initialization, as users had to create an account on the Fitbit website. For future applications, direct communication between the coaching system and activity trackers is preferred; however, this is likely not easy with commercially available trackers. 

We have decided to partly develop a native app for Android. A native app was necessary for implementing the location detection. New developments in standardization of location detection APIs and more advanced techniques for platform-independent development might result in a different choice, which could enlarge the potential user base. Another option would be to use the Google Fit API for getting information about the activity of users. Since its introduction, many systems and wearables directly integrate with this service.

### 3.3. Layout and Visual Design

Although the user interface of the app was designed in collaboration with a graphic designer, we put only minimal effort in its design. We did not receive any signs that this hampered the use of the app, but we imagine that following the design guidelines of the respective platform (Android and iOS) would improve the users’ perception.

### 3.4. Model-Based Reasoning

Some of the design choices in the personalization process described in Section 2.4 seem successful, but we identified opportunities for improvement in others. Our findings on these elements of the Active2Gether system are described below.

Based on anecdotic feedback, we conclude that determining the user’s awareness phase by comparing their actual behavior to their perception works well. Acknowledging the users’ awareness of the need to change is a useful way of tailoring the coaching messages.

In contrast, the suggestion of a coaching domain could be improved. The current approach is not very flexible, as the scores for active transport and stair walking are based on the characteristics of the significant locations that were identified via the intake questionnaire. Any physical activity related to these domains on other locations is ignored during the evaluation of the user’s behavior. Since that activity is not taken into account for either the actual behavior or the “ideal” behavior, this simplification should not distort the behavior scores. However, it is recommended to also take behavior on (or during transit to) other locations into account, to get a more complete picture of the user’s behavior. This could be achieved by using more adaptive behavior evaluation algorithms, which learn the user’s potential or ideal from past behavior, possibly in combination with other (web) sources.

Part of the selection of coaching messages is based on the simulations of a computational model. Although a preliminary validation of the model showed very promising results [21], the added value of the model in predicting the most effective coaching determinants still has to be evaluated. In theory, an adaptive approach can be used to learn the effect of specific (sets of) messages on a person’s behavior, which might lead to better suggestions for coaching determinants. The outcome of evaluating the model could for example lead to the decision to use personal and adaptive parameters in the computational model, or to take an entirely different approach (e.g., machine learning techniques).

### 3.5. Selection and Tailoring of Coaching Messages

The messages that people receive are very diverse, but sometimes still give the impression that they are redundant or not on topic. For a more detailed investigation of the user experience of the messages, see [8]. We have a number of suggestions for improvement.

First, the personal relevance of the messages could be improved. For example, the messages are only sent at specific times during the day, but the users’ physical location could be used to trigger messages as well. Furthermore, the selection of messages to be sent could be based on more complex combinations of information. For instance, combining the current location with the relevant floors on that location and the availability of stairs and the maximum number of stairs that a user is willing to walk. Incorporating these ideas would increase the context-awareness of the system. In addition, the messages should contain less trivial content in order to better fit the expectations of the target group.

Next, we implemented the selection of the message to be sent in such a way that the system sends the message that has been sent the longest time ago. However, if only a few messages are relevant, the users will still receive the same messages in a short time period. Therefore, it is important to adhere to a minimum amount of time between resending the same message. In addition, the interdependence or similarity between messages should be taken into account: if messages are different formulations of the same content, the system should be aware of that. If such improvements imply that a user does not receive any coaching messages for some period of time, the system could observe this and send a warning to the developers to make sure that this lack of relevant messages is noticed and possibly remedied.

Finally, the coaching messages could be improved by implementing a feedback mechanism. Instead of only being able to click “OK” to close a message, the user could rate the message, and this feedback could be used to further tailor the system.

### 3.6. Social Comparison

As explained in Section 2.6, the position of users in the ranking is based on their indicated preference. If people prefer upward comparison, they are shown users who perform better, and vice versa. This implies that users are always at the top or bottom position in the overview, irrespective of their performance. Although a study has shown that it matters to take the preferred direction of social comparison into account [27], the specific implementation might still allow room for improvement. A negative consequence of the current design decision is that users may become demotivated if they do not see any acknowledgment of their efforts. Therefore, a less strict selection of other users to show in the ranking might work better to motivate users through social comparison.

In addition, social comparison is more effective if you know the people you are comparing with. If users only have a few Facebook connections that are also using the system, it is likely that they mostly see anonymized other users that they do not know. Therefore, it might be better to allow adding connections via the system directly, or to invite friends to start using the system as well. Another option could be to select similar users (in terms of occupational status, home town, gender, age, etc.) to show in the ranking, and to show and emphasize these similarities in the design, in order to strengthen the perceived closeness to the other users.

Related to this is the issue that social comparison might not be equally beneficial for all types of people. For example, it is expected that patients and individuals managing chronic conditions are not so much interested in social comparison, but could benefit from social support. Although our system targets healthy individuals, in general it is important to take such personal characteristics into account when reasoning about the specific behavior change techniques that are applied by the system to the users.

A final consideration is of ethical nature. In the current implementation, it is easily possible that user A is shown the data of user B, but not vice versa. This means that individual reciprocity of information sharing is not ensured, which could cause objections from potential users. In that case, a more sophisticated selection mechanism should be developed, in which such reciprocity is maintained.

## 4. Conclusions and Future Work

In this paper, we have described the design of the Active2Gether coaching system in detail. The coaching system aims to encourage physical activity among young adults by combining evidence-based behavior change techniques with elements from modern (mobile) technology, such as location monitoring and model-based reasoning. 

The effectiveness of the system is currently being evaluated in a three-month trial with more than 100 participants between 18 and 30 years old. To determine the added value of the tailored messaging, a three-armed design has been chosen. One group uses the full version of the Active2Gether system, a second group uses the Active2Gether system but does not receive tailored messages, and the third group uses the standard website and app that belong to the Fitbit tracker. The participants start with an intake questionnaire that contains questions about their personal situation, their current exercise behavior and perceptions about physical activity. After three months, a similar questionnaire is sent out. The participants are also asked to wear a validated activity monitor in the first week of the intervention and after three months. This allows us to conclude whether using the system leads to a significant increase in physical activity. However, because several behavior change techniques have been employed in the system, it is difficult to identify *which* technique actually influences behavior. In the case a positive overall effect is found, we will further analyze which messages were sent to users to identify the contribution of specific techniques (focusing on specific determinants) on the behavior change. In addition, future research is needed with variants of the system in which only specific components (i.e., social comparison, self-monitoring and goal setting) are functional.

In the current paper, we have discussed the architecture and the implementation of the Active2Gether system. In addition, we have shared lessons learnt during the design, implementation and evaluation of the system, as well as recommendations for further development and improvement. We believe that these insights and the detailed description of the technological choices will prove helpful to designers and developers of healthy lifestyle interventions to produce effective and appealing coaching systems.

## Figures and Tables

**Table 1:** User categories based on objective/subjective evaluation.

No.	Objective	Subjective	User Category
1	Insufficient	Sufficient	The user is unaware that he/she is insufficiently physically active, and will be *educated* to increase this awareness.
2	Insufficient	Insufficient	The user is aware that he/she is insufficiently physically active, and will be *coached* to increase his/her physical activity level.
3	Sufficient	Insufficient	The user is sufficiently physically active, but still wants to be *coached* to increase his/her physical activity level.
4	Sufficient	Sufficient	The user is sufficiently physically active, and wants to maintain his/her physical activity level. This user will not be coached to increase his/her physical activity level, but only receive *feedback*.
